# The effects of menopausal uterine fibroids on the prognosis of endometrium cancer

**DOI:** 10.4274/tjod.galenos.2020.70104

**Published:** 2020-07-29

**Authors:** Önder Sakin, Ramazan Denizli, Zehra Meltem Pirimoğlu, Ali Doğukan Anğın, Muzaffer Seyhan Çıkman, Gökhan Gülyaşar

**Affiliations:** 1 İstanbul Dr. Lütfi Kırdar Kartal Training and Research Hospital, Clinic of Gynecology and Obstetrics , İstanbul, Turkey; 2 Arhavi State Hospital, Artvin, Turkey

**Keywords:** Endometrial carcinoma, uterine leiomyomas, myoma uteri, risk factors, post-menopause

## Abstract

**Objective:**

This study aimed to evaluate any potential associations between uterine leiomyomas and endometrial cancer.

**Materials and Methods:**

This is a retrospective study of 153 female patients who have been operated because of endometrial carcinoma in our hospital between 2012 and 2017. Data were collected from hospital records. Study participants were divided into two groups according to the presence and absence of leiomyomas. These two groups were compared in terms of histopathological adenocarcinoma type, nuclear and histological grades, disease stage, para-aortic lymph node involvement, and myometrial invasion. For data analysis, Statistical Package for Social Sciences 15.0 software package was used. Comparison between the two groups was made using the chi-square test, and each variable was tested with the Student’s t-test for statistical significance.

**Results:**

No statistically significant differences were found between the groups with respect to age, tumor type, myometrial invasion, nuclear grade, or histological grade (p>0.05 for all). A significant difference was found between leiomyomas presence and lymph node metastases. The lymph node metastases were more common in patients without uterine leiomyomas (20.55%) than in those with them (5%; p=0.004). Analysis using the Federation of Obstetrics and Gynecology stages for the presence of leiomyomas indicated that the mean stages were 1A and 1B in patients with and without uterine leiomyomas, respectively (p=0.002).

**Conclusion:**

Uterine leiomyomas did not adversely affect the prognosis of patients with endometrial carcinoma. Moreover, lymph node involvement was less common, and stages were lower in patients with leiomyomas.

**PRECIS:** Uterine fibroids in post-menopausal women do not have a negative impact on developing endometrium cancer.

## Introduction

The prevalence of endometrial carcinoma has a significant tendency to increase in developed countries and has become the most common cancer of the female genital tract in these countries^(1,2)^. Endometrial carcinoma is the most prevalent gynecological malignancy in the United States (USA), and 60,050 new cases of endometrial carcinoma with 10,470 endometrial cancer-related deaths have been reported in 2016^(3)^.

Based on the 2012 data, the estimated numbers of new cancer cases and cancer deaths worldwide were 14.1 million and 8.2 million, respectively^(4)^. Endometrial carcinomas accounted for 3.6% of new cancer cases and 1.8% of cancer deaths^(5)^. Of patients, 90% were over the age of 50 years, and the mean age was 63 years. However, 4% of patients diagnosed with endometrial carcinoma were under the age of 40 years^(6)^.

The prevalence of uterine leiomyomas is also high among women, and one out of every four women develops uterine leiomyoma. In a study conducted in the USA, leiomyomas were responsible for 46% of a total of 1.7 million hysterectomy procedures performed because of benign tumors^(7)^.

Every leiomyoma is derived from a single progenitor myocyte; therefore, each leiomyoma in the same uterus may have an independent cytogenetic origin^(8)^. The essential mutation underlying tumorigenesis remains unknown; however, karyotype abnormalities have been found only in 40% of leiomyomas. Several specific aberrations have been detected in chromosomes 6, 7, 12, and 14, which are associated with the direction and rate of tumor growth^(9)^.

A reduction is usually seen in the leiomyoma size during the postmenopausal period, and a new tumor development is usually rare during this period. Nearly all leiomyomas regress after menopause, which also result in the resolution of complaints associated with leiomyomas, such as bleeding and pain^(10)^. As physicians, we always favor conservative approaches in leiomyomas in premenopausal women for the abovementioned reasons and consider surgery as the last treatment option. During transition to menopause, we try to avoid surgery and follow up women with leiomyomas under medical treatment, if possible.

However, we do not know whether these leiomyomas, which are left uncontrolled, have any unfavorable effect on endometrial carcinoma, in the event that the patients develop this condition. Is it possible that leiomyomas left in place are associated with unfavorable prognostic factors if endometrial carcinoma occurs? Are there any associations between leiomyomas and factors that may impact survival? In this study, we aimed to investigate the potential relationships between endometrial cancer and fibroids.

## Materials and Methods

Patients with endometrial carcinoma who were operated in the obstetrics and gynecology clinics between 2012 and 2017 were included in this study. Histopathological studies conducted in the department of pathology of our hospital were retrospectively collected. A total of 153 patients were enrolled, and Ethics approval was obtained from the University of Health Sciences Turkey, Kartal Dr. Lütfi Kırdar Training and Research Hospital, Institutional Review Board (approval number: 2018/514/122/18, date: 30/01/2018).

In our study, we investigated whether the presence or absence of leiomyoma impacts the prognosis of endometrial carcinoma. Accordingly, patients with and without uterine leiomyomas were compared according to demographics, such as age, histopathological endometrial carcinoma type, nuclear and histological grades, disease stage, pelvic para-aortic lymph node (LN) involvement, and myometrial invasion depth. Potential associations between these parameters and the uterine leiomyoma size were also compared in subjects with uterine leiomyomas*.*

### Statistical Analysis

Continuous variables were presented as mean ± standard deviation, and categorical variables as percentages. The  Student’s t-test was used to compare normally distributed variables, and the results were interpreted within 95% confidence interval with a significance level of p<0.05. Statistical Package for Social Sciences software for Windows 15.0 was used for statistical analyses. The chi-square test was used for categorical variables.

## Results

A total of 153 patients were included in this study, and of whom, 135 had endometrioid endometrial carcinoma and 18 other types of endometrial carcinoma. Furthermore, 80 had concomitant leiomyomas, with 64 having tumors less than 3 cm and 16 larger than 3 cm. Myometrial invasion was less than one-half in 97 patients and more than one-half in 56. LN metastasis assessment revealed that 134 patients were LN positive and 19 negative. Of all patients, 52.99% were under the age of 60 years, and 88.24% had endometrioid tumors. Leiomyomas were present in 80 patients (52.29%), and tumors were less than 3 cm in size in 64 (80%) of these patients. Demographic characteristics of these patients are shown in Table 1.

Comparisons of age, tumor type, invasion, and LN metastases by the presence of leiomyomas revealed a significant association between leiomyoma presence and LN metastases. LN metastases were significantly more common in patients without leiomyomas (20.55%) than in patients with leiomyomas (5%; p=0.004). No significant associations were found between leiomyoma presence and age, tumor types, or invasion (p>0.05 for all; Table 2).

The stage comparisons by leiomyoma presence revealed that the mean stages were 1A and 1B in patients with and without uterine leiomyomas, respectively (p=0.002). The comparisons of nuclear and histological grades by leiomyoma presence did not reveal any significant associations (p>0.05).

The comparisons of age, tumor type, invasion, and LN metastases by leiomyoma size indicated that the prevalence of LN metastases was significantly higher in patients with leiomyomas larger than 3 cm (18.75%) than in those with less than 3 cm (1.56%; p=0.005). No significant associations were found between leiomyoma size and age, tumor type, and invasion (p>0.005 for all; Table 3).

The comparisons of stages by leiomyoma presence did not reveal any significant associations between the leiomyoma size and disease stages (p>0.05), and the assessment of nuclear and histological grades by the leiomyoma size did not reveal any significant associations either (p>0.05).

The assessments of nuclear and histological grades by age in overall patients revealed a weak correlation. Nuclear (r=0.184, p=0.023) and histological grades (r=0.193, p=0.017) increased as the age increased (Table 4).

When fibroid presence was examined by regression analysis, it was not associated with age, tumor type, and invasion, and fibroid presence was found to decrease the risk of LN metastasis by 3.89 times (Table 5).

## Discussion

Important factors for recurrence and survival in endometrial cancer are age, tumor type, grade, myometrial invasion, and LN metastasis. Similarly, the type of operation may vary after surgical staging, although sometimes, it is sufficient to perform a type 1 hysterectomy, and in some cases, radical surgery such as pelvic para-aortic LN dissection and omentectomy may be required^(11)^. In this study, we compared the prognostic factors such as age, tumor type, grade, amount of myometrial invasion, and LN metastasis by dividing endometrium cancer patients into two groups. According to the presence or absence of fibroids or adenomyosis, this study aimed to investigate whether fibroids have a negative effect on endometrial cancer.

The relationship between fibroid presence and endometrial cancer was first suspected by Giammalvo in 1958. He reported an increase in the frequency of adenomyosis in women who were operated for endometrial cancer^(12)^. In a study by Greenwood in 1976, adenomyosis was seen in 19.4% of 175 patients operated for endometrial cancer, 20.5% in 254 endometrial hyperplasia, and 16.7% in 203 prolapse cases and found to be statistically similar^(13)^. On the other hand, 136 patients with endometrial cancer and 222 with hysterectomy for uterine prolapse were examined in another study. The incidence of adenomyosis was found to be higher in cases of endometrial cancer, especially in women with postmenopausal endometrial cancer, reporting 1.5-2 times more adenomyosis and uterine fibroids^(14)^. A study in Turkey examined hysterectomy specimens from those operated because of benign conditions and found that the incidence of fibroids and adenomyosis was 21.6%^(15)^. When 130 endometrial cancers were examined in a study by Menczer et al.^(16)^, the frequency of fibroids was 56.9%. In our study, the presence of fibroids or adenomyosis was 52.29% in patients with endometrial cancer and 2-2.5 times higher than the general population, and this was found to be consistent with previous studies.

The degree of myometrial invasion is one of the most important parameters in surgical staging. According to the research of  Taneichi et al.^(17)^ with 362 endometrial cancers in 2014, myometrial invasion is deeper in the case of adenomyosis and fibroids and two times deeper in myometrial invasion. They claimed that even with the presence of adenomyosis, recurrence and mortality did not increase, and it may lead to deep myometrial invasion. This claim was not supported in other studies^(16,18,19,20)^.

In a study by Studzinski et al.^(18)^, 136 cases were examined, and fibroids did not affect the stage and surveillance of endometrial cancer patients. Similarly, 130 patients with endometrial cancer were examined in another study; myometrial invasion, LN invasion, and metastasis presence and pathology did not change the stage^(16)^. Gizzo et al.^(19)^ examined 289 endometrial cancers in their study and reported that in the presence of adenomyosis and fibroids, the stage is lower; myometrial invasion and LN metastasis were also less common. In our study, even if not statistically significant, the presence of deep endometrial invasion in the presence of fibroids was less common. Similarly, patients who have myoma were diagnosed at an earlier stage.

Metastasis in endometrial cancer usually occurs by lymphatic route. LN metastasis is crucial for disease recurrence and surveillance^(20)^. According to a study, with the presence of adenomyosis and fibroids, LN metastasis has been seen less commonly^(21)^. A study by Koshiyama et al.^(22) ^in 2004 divided 179 endometrial cancer patients into four groups, namely, adenomyosis, endometriosis, fibroids, and endometrial cancer alone. Patients were reported to be diagnosed at an earlier age in the presence of adenomyosis and fibroids. They reported that this may be due to the patients’ earlier admission to hospital because of additional symptoms. In our study, in the presence of fibroids, LN metastasis was detected less frequently and was found to be consistent with the literature.

In our study, we also divided the patients into two groups: 3 cm below and above. We classified the patients according to the fibroid size and compared the variables, including age, tumor type, myometrial invasion, and LN metastasis. When we examined the literature, we could not find any study examining the relationship between fibroid size and endometrial cancer. In our study, no significant relationship was found between fibroid size and age, tumor type, and myometrial invasion. Thus, statistically similar results were observed. LN metastasis was found to be higher in patients with fibroid size over 3 cm, and this was statistically significant. Since the total number of patients with fibroids and LN metastasis is very small, we believe that larger randomized studies are needed.

### Study Limitations

This study has limitations that should be acknowledged. Firstly, the number of patients is low, especially in some subgroups. Secondly, there is a lack of data on long-term results of patients. Finally, we had difficulty comparing our study with other studies in the literature because there were not enough studies.

## Conclusion

Uterine leiomyomas did not adversely affect the prognosis of patients with endometrial carcinoma. In addition, LN involvement was seen less, and stages were lower in patients with leiomyomas.

## Figures and Tables

**Table 1:**
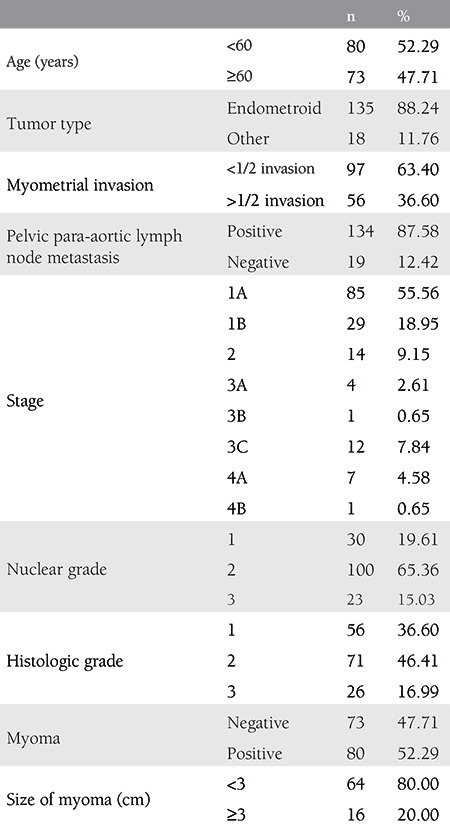
Demographics of patients

**Table 2:**
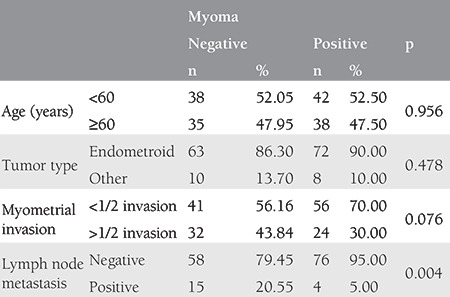
Comparison of age, tumor type, invasion, and lymph node metastasis according to the presence of myoma

**Table 3:**
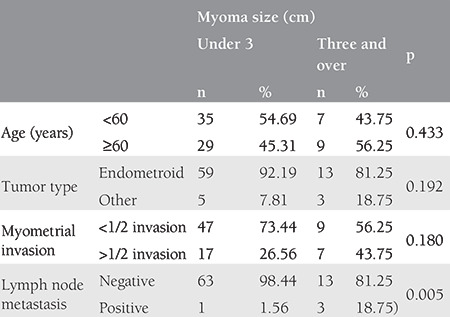
Comparison of age, tumor type, invasion, and lymph node metastasis according to the size of myoma

**Table 4:**
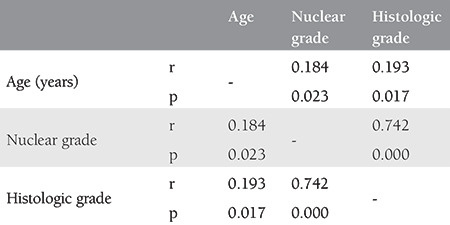
Correlation between age and nuclear and histological grades in all patients

**Table 5:**
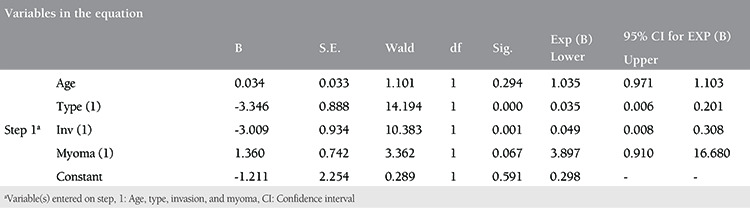
Presence of fibroids is examined by regression analysis
